# On the Process and Product Fingerprints for Electro Sinter Forging (ESF)

**DOI:** 10.3390/mi10040218

**Published:** 2019-03-27

**Authors:** Emanuele Cannella, Chris Valentin Nielsen, Niels Bay

**Affiliations:** 1 Department of Mechanical Engineering, Technical University of Denmark, DK-2800 Kongens Lyngby, Denmark; cvni@mek.dtu.dk (C.V.N.); nbay@mek.dtu.dk (N.B.); 2 IPU, Instituttet for Produktudvikling, DK-2800 Kongens Lyngby, Denmark

**Keywords:** Electro sinter forging, resistance sintering, electrical current, fingerprints

## Abstract

Electro sinter forging (ESF) represents an innovative manufacturing process dealing with high electrical currents. Classified in the category of electrical current assisted sintering (ECAS) processes, the main principle is that Joule heating is generated inside the compacted powder, while the electrical current is flowing. The process is optimized through the analysis of the main process parameters, namely the electrical current density, sintering time, and compaction pressure, which are also evaluated as process fingerprints. The analysis was conducted on commercially pure titanium powder. Small discs and rings were manufactured for testing. The influence of the process parameters was analysed in terms of the final material properties. The relative density, microstructures, hardness, and tensile and compressive strengths were analysed concerning their validity as product fingerprints. Microstructural analyses revealed whether the samples were sintered or if melting had occurred. Mechanical properties were correlated to the process parameters depending on the material. The different sample shapes showed similar trends in terms of the density and microstructures as a function of the process parameters.

## 1. Introduction

Among several manufacturing processes, sintering represents a well-known solution based on powder consolidation via thermal heating. Ceramic objects were the first examples of sintered components, while metal sintering is relatively new. The first literature containing sintering is dated from 1829 [1]. In that case, platinum was attempted. Since that time, researchers have mostly focused on new materials and technologies. Conventional sintering includes two steps, namely (i) compaction of the sintering powder with the generation of the green body, and (ii) heating in a sintering furnace. The material increases its density by thermal energy, enabling a reduction of the free-surface energy as described by the Gibbs equation [2]. There are three main contributions in achieving the energy reduction, i.e., external pressure, particles geometry, and chemical reactions [3]. The main advantage of sintering is the possibility of manufacturing complex shapes with a large reduction of scrap material. Furthermore, the internal structure is less affected by the process nature, as it appears in conventional hot and cold forging. However, the high temperatures involved in sintering can generate some problems concerning creep, grain growth, and oxidation [4]. To reduce these effects, new sintering technologies have focused on reducing the time inside the furnace. Hot pressing (HP), where the two main steps are carried out simultaneously, represents an alternative to conventional sintering. The green body is heated inside the sintering die while the compaction pressure is maintained during the whole process. The thermo-mechanical field enables an increased densification rate [5]. Higher densities are achieved because the micro-porosities and elastic spring back are limited by the pressure action [6] and increased compact strength [7]. Later developments introduced a new heating approach based on an electrical current and Joule heating. To achieve high temperatures in a very short time, the electrical current flows through the green compact and/or the die. The pressure is maintained during the process as in HP. In general terms, it is identified as field-assisted sintering technology (FAST) [8]. As described by Grasso et al. [9], the term, “field”, may identify different sources, e.g., mechanical, electrical, or electro-magnetic sources. Thus, electrical current assisted sintering (ECAS) is suggested as a subgroup to identify those processes where the electrical current is used for heating. The classification is done as a function of the electrical current waveforms and sources, loading methods, discharge time, and maximum peak current. In the case that electrically non-conductive powders are used, the electrical sintering provides the heating of the die. The approach is therefore comparable to HP, but the heating rate is increased by utilizing Joule heating. One of the most representative and oldest names is spark plasma sintering (SPS). The die and electrodes are usually made of graphite, enabling the current to flow within both the die and green compact, in which case it is conductive. The high density achieved is justified by the idea of plasma formation. However, no proof of plasma has been shown experimentally [10]. In the case of conductive powders, the electrical current flowing inside the green compact enables direct heating of the samples. The achieved high density is therefore a consequence of the complex electro-thermo-mechanical field, generating several phenomena among the particles, e.g., dielectric breakdown of the oxide layers and pinch-effects [11]. Alternating current, direct current, and capacitor discharge technologies are used [12]. The different current profiles result in different heating rates, involving “fast” processes, above 0.1 s, and “ultrafast” processes, below 0.1 s. The electrical current density is therefore set with lower values in the “fast” case, below 10 A/mm^2^, than in the “ultrafast” case, above 100 A/mm^2^ [9]. For example, flash sintering enables such reduced times by using high capacitor discharges [13]. The pressure can be kept constant or altered while processing, which is the case of electro sinter forging (ESF) [14].

Generally, the main process parameters are the electrical current, time, and compaction pressure. Several conductive powders have successfully been sintered with those processes, e.g., gold [15], copper [16], titanium [17], and melt spun magnetic powders [18]. However, the process limits are related to the material and machine properties. This requires a careful trade-off of the process parameters in connection with the final product characteristics, defined as process and product fingerprints.

By applying the “ultrafast” ESF principle, the present research focuses on the analysis of the effects derived from different process settings, defined as process fingerprint candidates, for ESF. The final quality of the components is affected by the electrical current, pressure, and time. The process window is limited by the required energy to ensure adequate consolidation of the particles and yet is sufficiently low enough to avoid any melting of the material. In the case of an alternating current, the electrical sine wave influences the results, because of the high current amplitudes. Furthermore, programming the alternating current machine is more complicated than the direct current machine. All sintered parts are analysed in terms of density. Mechanical properties are evaluated in terms of hardness and tensile and compressive strengths. Micrographs are made to identify and compare the achieved microstructures. The validity of these properties as product fingerprints is investigated based on their empirical connections with the process parameters. Fingerprints are found when unique input/output correlations are established. 

## 2. Materials and Methods

**Figure 1:**
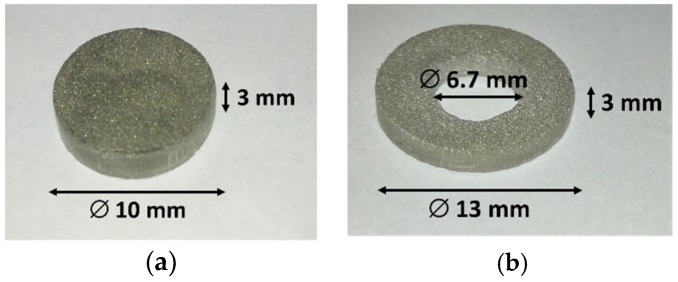
Examples of sintered titanium samples by (**a**) a disc and (**b**) a ring.

**Figure 2:**
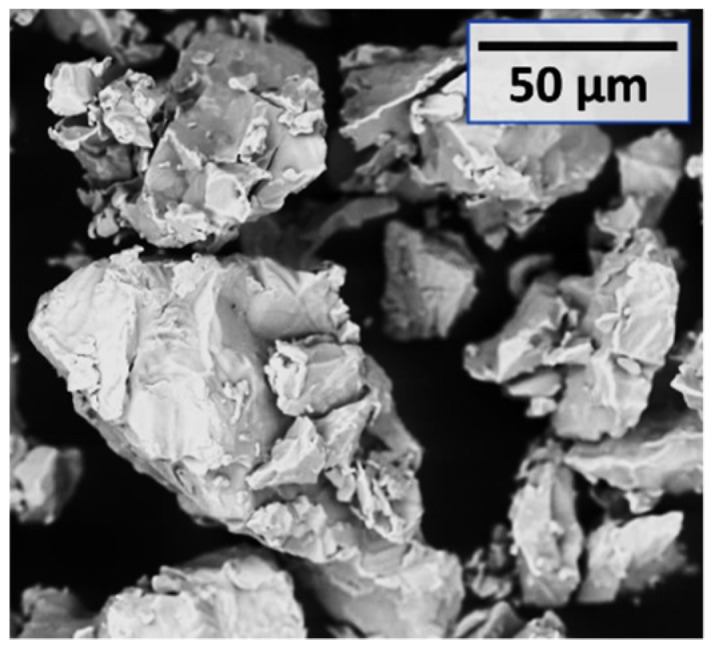
Scanning electron microscope (SEM) backscattered electron detector (BSD) image of the titanium powder used for sintering.

Disc- and ring-shaped specimens were manufactured during the experiments (Figure 1). An axisymmetric design facilitated the investigation of the sintering process by limiting the problems related to thermal gradients. Rings were produced to analyse the results obtained with an additional tool contact surface, which influences the obtained properties. The tested material was commercial pure titanium (GoodFellow Inc., Huntingdon, UK), which is electrically conductive and has a melting temperature of 1668 °C [19]. A neodymium alloy, namely NdFeBCo (Magnequench, Tianjin, China), suitable for permanent magnets, was investigated in parallel research by the present authors (ongoing work by the present authors). The titanium raw powder is shown in Figure 2 by scanning electron microscope (SEM) backscattered electron detector (BSD) images. The sintered discs have an external diameter of 10 mm and a thickness of 3 mm. The rings were designed to have a comparable cross-sectional area and therefore similar electrical current density with equal nominal electrical values. They have an outer diameter of 13 mm, an internal diameter of 6.7 mm, and a 3 mm thickness.

**Figure 3:**
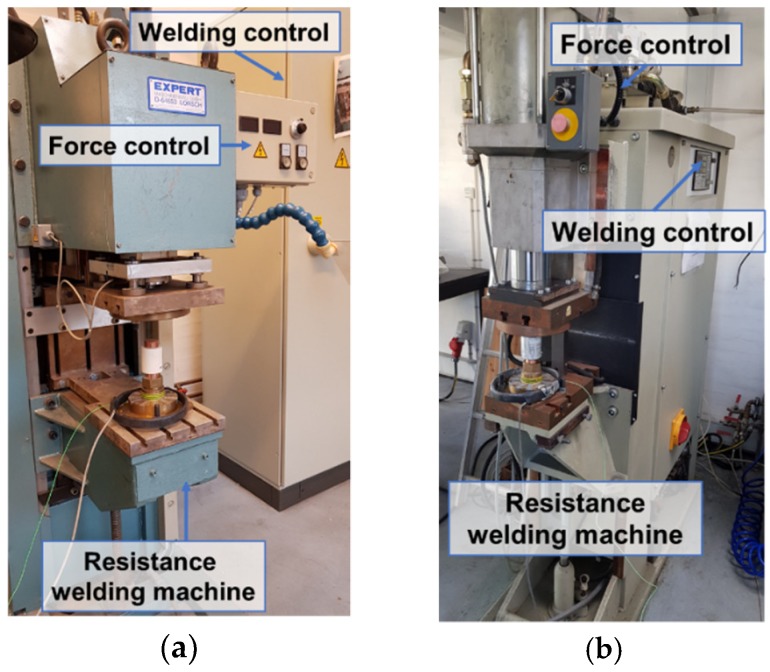
Resistance welding machines used for sintering, (**a**) middle frequency direct current (MFDC) and (**b**) alternating current (AC) resistance welders.

The ESF experiments were carried out on two different resistance welding machines (Figure 3), i.e., middle frequency direct current (MFDC) machine from Expert (Expert Maschinenbau GmbH, Lorsch, Germany) and an alternating current (AC) machine from Tecna (Tecna, Castel S. Pietro Terme, Italy), in order to test different current profiles with comparable levels of total electrical energy. The two machines are equipped with different mechanical systems. The MFDC machine is hydraulically operated and has disc-springs for the follow-up of the compaction force, while the AC machine is pneumatically operated.

**Figure 4:**
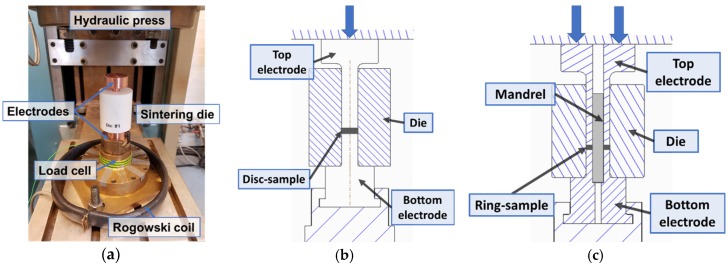
Pictures showing the (**a**) tool system used for electro sinter forging, (**b**) tool design for the disc, and (**c**) tool design for the ring.

The tool system was made by an electrically insulated die made of aluminium oxide and conductive punches/electrodes made of copper (Figure 4a). The two different tool designs shown in Figure 4b,c were used for discs and rings, respectively. The disc tool design consists of a cylindrical die with an inner hole. The ring tool design has an additional, removable alumina mandrel radially aligned by the two hollow electrodes. The mandrel prevents inwards flow of the ring specimen during ESF. After each ESF, the mandrel is removed and the ring is ejected from the die.

## 3. Process and Product Fingerprints

Process and product fingerprints are considered as unique inputs and outputs for a manufacturing process. The definition of fingerprints is important to control and monitor the ESF process and to guarantee product quality. The following sections present specific process and product fingerprints for ESF of the discs and rings.

### 3.1. Process Fingerprints

**Figure 5:**
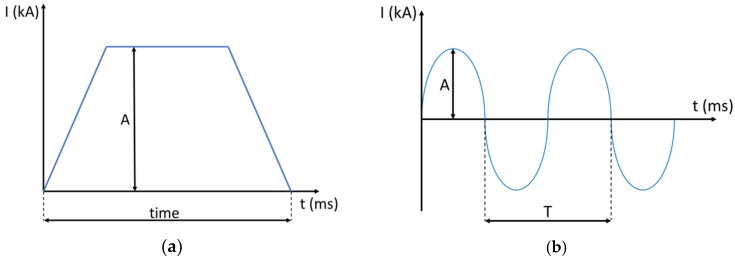
Typical electrical profiles in the case of (**a**) direct and (**b**) alternating currents.

Two different resistance welding machines were used. They differ in the generated current profiles, which are direct (Figure 5a) and alternating electrical currents (Figure 5b).

**Figure 6:**
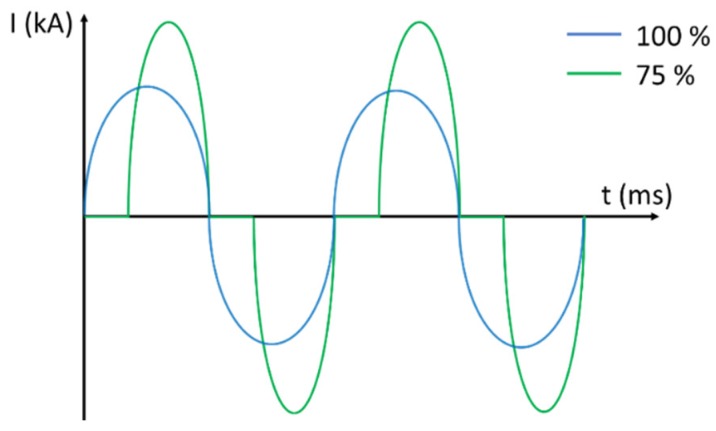
Illustrated examples of alternating current waves for different conduction angles, 75% and 100%.

In the case of the MFDC (Figure 5a), the nominal value, *A*, is set on the machine, while on the AC machine, the *RMS* value is entered. *RMS* indicates the root-mean-square value of the electrical current profile, which in the case of a 180° conduction angle, would be estimated as:

(1) $$RMS=\;\frac{A}{\sqrt{2}}$$

where *A* is the current amplitude (Figure 5b). For smaller conduction angles, the relation becomes more complicated because the wave form changes from the ideal sinusoidal form. The conduction angle represents the amount of time in which the current flows. Figure 6 shows the current profile for two experiments running with different conduction angles, 75% and 100%, but the same *RMS*. Figure 6 shows how the required amplitude for a given *RMS* depends on the conduction angle. 

The monitoring of the current was performed in-line by using a Rogowski coil (Tecna) as shown in Figure 4a. Data were saved for post processing and the electrical current was converted into an average current density. The load was in-line measured by using a piezoelectric load cell (Kistler, Winterthur, Switzerland) placed inside a brass cage as shown in Figure 4.

**Figure 7:**
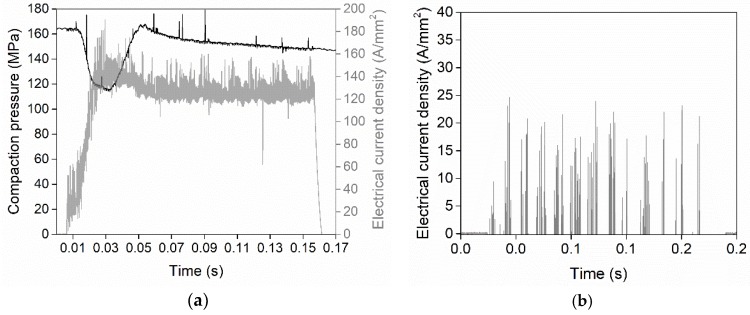
Typical MFDC diagrams when the sintering process is (**a**) correctly carried out and (**b**) aborted.

In-line monitoring of both the electrical current and load is important to verify that the process is performed correctly. If the electrical resistance of the powder compact is too large, no current is delivered due to the machine safety control, and sintering does not take place. This information was immediately available from the measured process parameters as shown in Figure 7. Figure 7a shows a typical diagram of the compaction pressure and the current density for a successful case, whereas Figure 7b shows an example in which the current is not delivered properly. In the successful case (Figure 7a), there is also a characteristic pattern between the pressure and the current density. The compaction pressure decreases when the current raises due to softening of the material and stabilizes shortly after the mechanical follow up from the machine.

The voltage and temperature are parameters that can be considered as process fingerprints. Measuring the voltage may help in monitoring the density achieved while sintering. The model considers the variation of the electrical resistance of the material due to the increasing densification of the sample. According to Montes et al. [20], the electrical resistivity, *ρ_e_*, of a metal powder aggregate, at room temperature, can be expressed as:

(2) $$\rho_{e}=\;\rho_{0}{\left(1-\theta_{r}\right)}^{-r}$$

where *ρ*_0_ is the electrical resistivity at bulk density and room temperature, *θ_r_* is the instantaneous porosity of the compact, and *r* is a resistivity exponent defined as:

(3) $$r=\;1+{\left(1-\theta_{m}\right)}^{4/5}$$

where *θ_m_* is the starting porosity before sintering. The electrical resistivity, *ρ_t_*, is also influenced by the temperature according to the following formula [21]:

(4) $$\rho_{t}=\;\rho_{e}\left[1+\alpha \left(T-T_{0}\right)\right]$$

where *ρ_e_* is the aforementioned electrical resistivity at room temperature for a metal powder aggregate, *α* is the temperature coefficient of resistance, and *T* and *T*_0_ are the instantaneous and room temperatures, respectively. Knowing the temperature also helps in understanding the process limits in terms of the maximum electrical energy. The maximum temperature should not go beyond the melting point, since the subsequent rapid cooling results in a dendritic structure, which is unsuitable for some applications due to generations of micro-voids.

In the present work, both the voltage and temperature were difficult to measure because of technological and process limits. For the voltage, attempts resulted in an increasing noise being generated in the data acquisition system, which affected the electrical current measurements. Temperature was an issue for the process characteristic itself. Very short sintering times and a closed die configuration made the conventional instruments, e.g., thermocouples and pyrometers, unsuitable for the purpose. Attempts were made by numerical simulations [22].

### 3.2. Product Fingerprints

To achieve properties as good as those in conventional manufacturing processes, the final density must be close to the bulk one. The internal porosities influence the achieved mechanical strength and increase the risk of crack propagation. However, for some advanced applications, e.g., biomedical products, a certain porosity is preferred [23]. For this reason, density is the most fundamental product fingerprint. The estimation of density was based on individual measurements of mass and volume. The mass was easily estimated by using a precision balance (Sartorius GmbH, Göttingen, Germany), while the volume was analysed by Archimedes’ method [24] and 3D volume reconstruction. The 3D volume was estimated by using metrology software (GOM GmbH, Braunschweig, Germany). Compatible density estimations were achieved by the authors [25]. Although volume reconstruction requires a long measuring time, the sample was not influenced by the uncertainty produced by Archimedes’ method in the case of highly porous samples, where liquid penetration affects the result by underestimating volumes and therefore overestimating densities. Precautions were taken by embedding the porous specimens in a waterproof resin.

**Figure 8:**
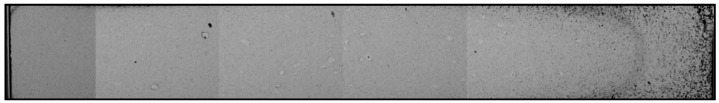
Example of a diametrical disc cross-section ready for local porosity investigation. This sample is made of titanium and sintered by MFDC. The compaction pressure is 170 MPa, the sintering time is 150 ms, and the electrical current density is 178 A/mm^2^.

The average porosity can be estimated by:

(5) $$\theta =\;1-\frac{d}{d_{0}}$$

where *d* and *d*_0_ are the sample and bulk densities, respectively. A detailed porosity analysis was achieved by cutting the specimen at a diametrical cross-section. After being ground and polished, the surface was investigated by optical analysis (Figure 8). An image analysis software was used to compute the porosity for the investigated area, by counting the number of black and white pixels representing porosity and material, respectively. Computed tomography is also a powerful instrument for porosity investigation [26].

The microstructure evaluation gives information regarding the sintering effect on the powder particles. The appearance of difference phases can vary as a function of the sintering parameters. In the case of ESF, the analysis of microstructures gave indications of the possible melting of the material. If the melting temperature was not reached, the micrographs clearly showed the bond interface between the particles. When high electrical currents were applied, the high temperatures exceeded the melting point of the sintered material. The achieved structures were typical of a melted material, as will be shown in Section 4.2. Microstructures were also used to investigate possible contaminations and oxidations of the material.

**Figure 9:**
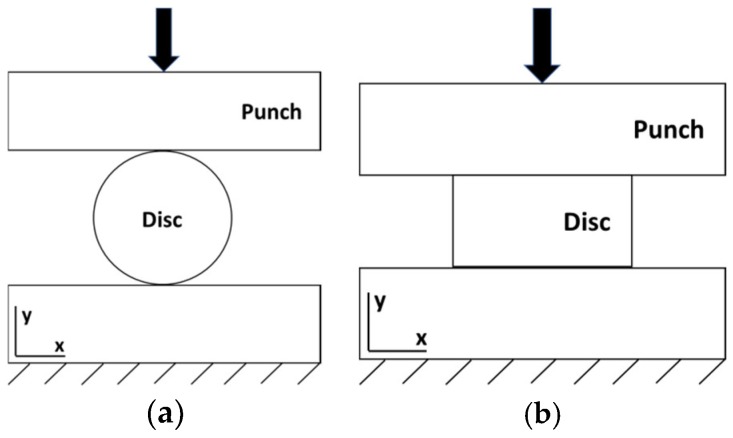
Schematic setups of the (**a**) indirect tensile test and (**b**) compression test.

Mechanical properties were investigated in terms of hardness, tensile, and compressive strengths. Indirect tensile tests (IDT) [27] were conducted for the discs and compression tests were carried out for both the discs and the rings. A 600 kN hydraulic press (Mohr & Federhaff AG, Manheim, Germany) was used for both approaches. As shown in Figure 9a, the IDT requires compression of the sample perpendicular to its centre axis to generate a tensile fracture at the centre of the disc specimen by tension perpendicular to the compression direction. Linear elastic behaviour is assumed until fracture occurs in the IDT. Compression tests were performed as illustrated in Figure 9b.

Hardness tests were performed according to the Vickers method given by ISO 6507 [28]. A pyramidal indenter was applied on the polished surface. Micro Vickers equipment was used for the purpose, with standard HV0.1 (100 g) and 50× optical lens (Olympus, Tokyo, Japan).

In addition, further product fingerprints can be selected based on the specific product. Geometrical and dimensional features can be important fingerprints to be monitored and related to the process settings. For example, in the case of axisymmetric components designed for rotational applications, e.g. rotors, the eccentricity is a natural product fingerprint.

## 4. Results

### 4.1. Density Analysis

**Figure 10:**
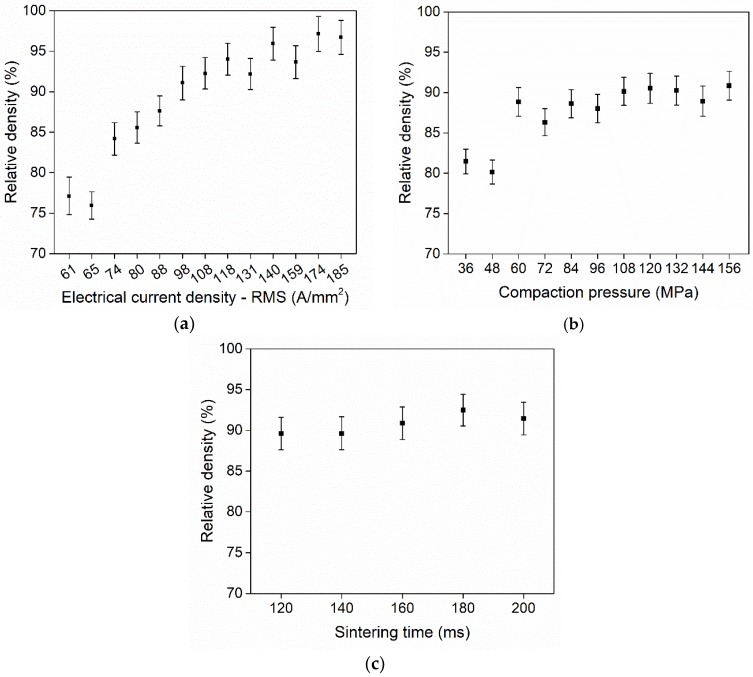
Density variation for titanium discs sintered in the AC machine as a function of the (**a**) electrical current density (84 MPa, 100 ms), (**b**) compaction pressure (98 A/mm^2^, 100 ms), and (**c**) sintering time (98 A/mm^2^, 94 MPa).

Titanium discs were sintered on both the MFDC and AC machines to evaluate the influence of the applied current profile. In both cases, an evaluation of the parameters with the most influence and an optimization of the process parameters were carried out. Figure 10 shows such a study based on the AC machine. The relative densities of the obtained samples are shown for various process settings, and in agreement with Joule’s law of heating, the electrical current was found to be the most influencing process parameter. The parameter ranges were (i) 61–185 A/mm^2^ *RMS* for the electrical current density, (ii) 36–156 MPa for the compaction pressure, and iii) 120–200 ms for the sintering time, which is entered as the number of cycles with a 20 ms period (50 Hz). The error bars in Figure 10 represent the measurement uncertainty estimated by taking into account the resolution of the balance and 3D scanner for measuring the mass and volume, respectively. The error propagation formula was applied based on the density formula and a coverage factor, k = 2, at a confidence level of 95%, according to the ISO GUM [29]. Figure 10a shows the obtained relative density as a function of the electrical current density. The density increases with the increasing current until around 140 A/mm^2^, where it stabilizes. Internal melting of the samples occurred for the highest current densities, as shown by the micrographs in Section 4.2. Until 60 MPa, the compaction pressure increased the density (Figure 10b). Hereafter, the compaction pressure did not influence the density. The sintering time did not affect the final density in the range of 120–200 ms (Figure 10c).

Similar trends were obtained when sintering titanium (ongoing work by the present authors) and magnetic (ongoing work by the present authors) discs in the MFDC machine. As a general conclusion, sintering was possible in both of the machines. However, the MFDC is more controllable than the AC, which is more prone to melting the samples due to the high current amplitudes, at the same electrical energies. 

**Figure 11:**
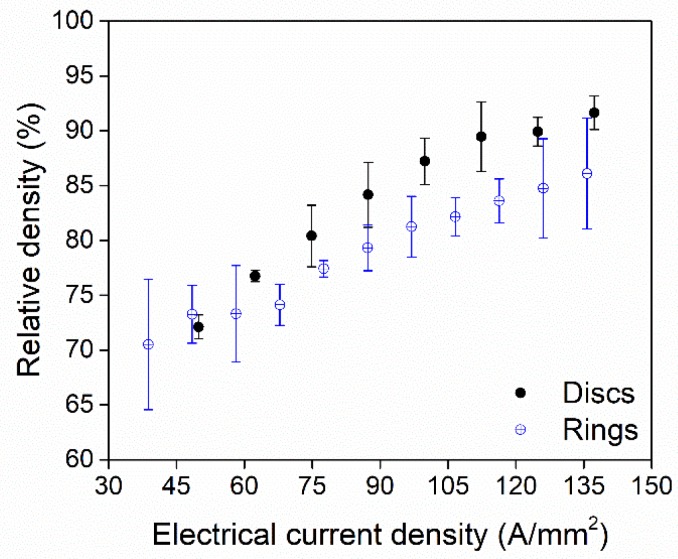
Comparison between the achieved densities for titanium discs and rings produced in the MFDC machine as a function of the electrical current density (150 ± 20 MPa, 150 ms).

The titanium rings were only sintered in the MFDC machine to avoid further complications from the AC current profile. Figure 11 shows the obtained relative densities for both the disc and ring-shaped samples as a function of the applied current density. The error bars again show the measurement uncertainties, which are calculated as described above in relation to Figure 10. As noted in Section 2, the axial cross-section of the disc and the ring were chosen to have comparable areas. The heights are also the same, and therefore, the initial resistance and expected current density are also comparable. The results showed how the contact area between the rod and compacted powder introduced larger thermal gradients, resulting in a lower density in the ring than in the disc for the same current density. This is more pronounced at high electrical currents, where the thermal gradients are steeper. Furthermore, the cross-sectional analyses in Section 4.2 show the poorer distribution of porosities in the ring samples due to the additional thermal gradient on the inside of the ring. 

### 4.2. Microstructures

**Figure 12:**
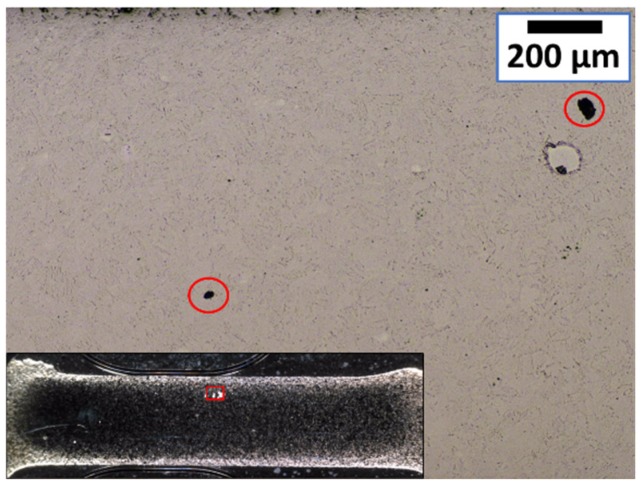
Micrograph showing examples of closed porosities for a titanium sample sintered by MFDC at 115 A/mm^2^ for 150 ms under a 170 MPa compaction pressure.

Microstructural observations give an important overview of the particle bonding effect after sintering. The sintering limit can be defined as the maximum total energy that can be applied before melting. Depending on the application, melting can be accepted or not. However, in the case of melting, closed porosities as found in Figure 12, can be generated and therefore influence the final properties of the material.

**Figure 13:**
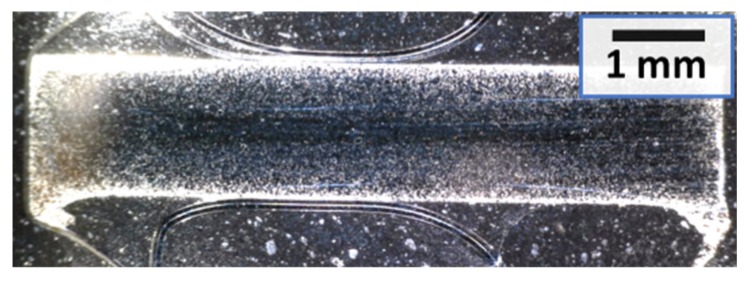
Optical picture showing the diametrical section of a titanium disc sintered by MFDC at 165 A/mm^2^ for 150 ms under a 170 MPa compaction pressure. An internal fully dense core (dark) and a porous outer part (bright) can be seen.

Furthermore, it is possible to note how the most heated zone is the core of the sample (Figure 13). This can be explained by the cooling of the tools on the surface and the larger electrical resistance in the core before sintering. The reason for the latter is the compaction itself, which is not homogeneous. The result is a fully dense core surrounded by porous material.

**Figure 14:**
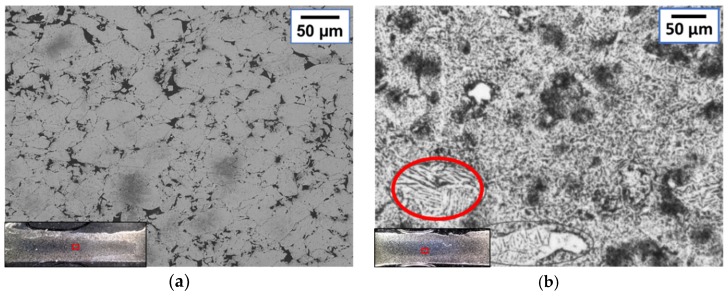
Micrographs showing the achieved microstructures for titanium samples sintered at (**a**) 76 A/mm^2^ MFDC for 150 ms at a compaction pressure of 170 MPa and (**b**) 76 A/mm^2^ *RMS* AC for 100 ms at a compaction pressure of 82 MPa.

Micrographs of samples sintered with the AC machine were compared with the samples sintered with MFDC. The results highlighted the difficulty in controlling the alternating current in terms of energy peaks due to the high amplitudes. The appearance of melted material was found at 76–89 A/mm^2^ in the case of AC, while 115 A/mm^2^ could be applied before melting with the MFDC. In the pictures shown in Figure 14, the comparison between titanium samples made by MFDC and AC is shown. At 76 A/mm^2^, MFDC samples (Figure 14a) have a typical sintered microstructure with bonded particles. Conversely, in Figure 14b, dendritic structures typical of a melted and rapidly solidified material are shown in a sample made by AC. In the latter case, the high amplitudes resulted in current density peaks of 188 A/mm^2^, which is 2.5 times the *RMS* value and the corresponding current density when using MFDC.

**Figure 15:**
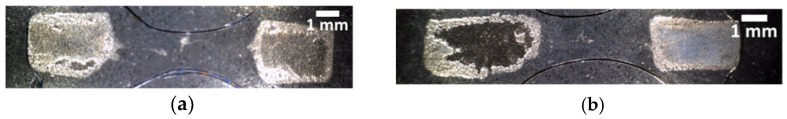

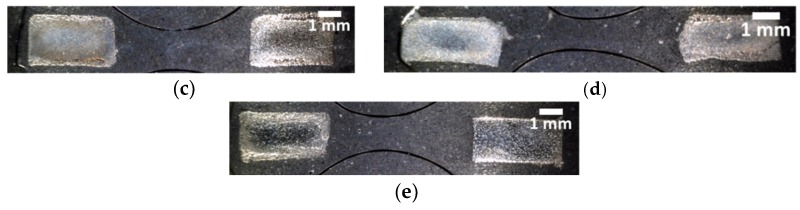
Optical images showing the diametrical sections obtained for sintered titanium rings at 133 MPa, 150 ms and (**a**) 60, (**b**) 82, (**c**) 103, (**d**) 123, and (**e**) 144 (A/mm^2^).

**Figure 16:**
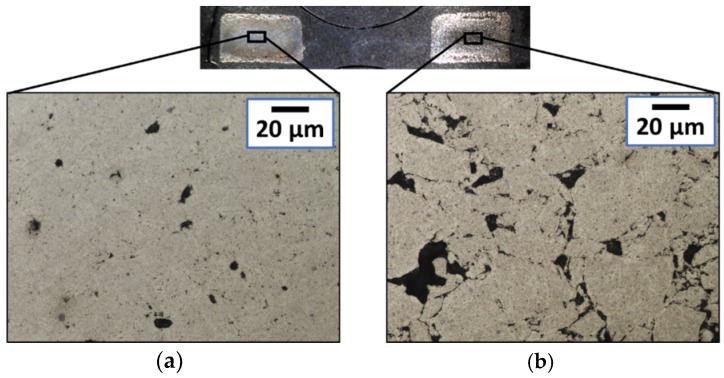
Optical micrographs showing the detailed microstructures achieved for a titanium ring sintered at 133 MPa, 150ms, and 103 A/mm^2^. The differences between the two sides are clear by having (**a**) a dense core with micro-voids and (**b**) bonded particles not completely densified.

When sintering titanium rings, the structures and density become asymmetric. This was observed when samples were diametrically cut and embedded into resin (Figure 15). The achieved structures suffered from thermal and pressure gradients created between the two sections of the same diametrical cut. The main reason is the inhomogeneous starting density of the green body after the initial compaction. The central mandrel constituted an obstacle to the reallocation of particles during compaction. Furthermore, according to the theoretical model described by Equations (2) and (3), an increase in electrical resistivity is seen with an increasing porosity. This provoked inhomogeneous Joule heating, inducing a thermal gradient. Figure 16 shows the influence on the microstructure in one example. In Figure 15e, the geometry is also affected, showing a well-shaped rectangular section on one side (right) and a slightly deformed one on the other side (left).

Magnetic rings broke during ejection due to their brittleness. The structure was not strong enough to withstand ejection. The problems experienced when sintering rings may be reduced by limiting thermal dissipation to the sintering tools. Pre-heating of the die and electrodes could also be investigated.

### 4.3. Mechanical Properties

Hardness, tensile, and compressive tests were performed to evaluate the mechanical quality of the sintered samples.

**Figure 17:**
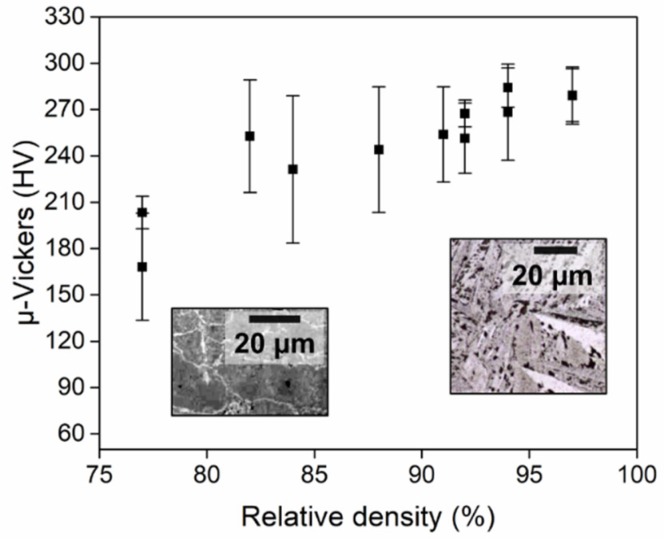
Hardness measured for titanium samples sintered by AC as a function of the relative density. Each point represents a different sample, and corresponding error bars refer to measurement uncertainties. Microstructures are distinguished for low and high densities.

The hardness measurements are shown in Figure 17 for titanium discs sintered by AC. Seven indentations were performed at the core with about a three diameters distance to avoid any influence from previous indentations. The average values and standard uncertainties were estimated for each analysed sample. The expanded uncertainty was evaluated with a coverage factor of *k* = 2 at a confidence level of 95%, according to the ISO GUM [29]. Figure 17 shows how the hardness is influenced by the achieved microstructures. The bonded microstructures seen for low relative densities (<80%) showed a lower average hardness than in the case of high relative densities (>80%).

**Figure 18:**
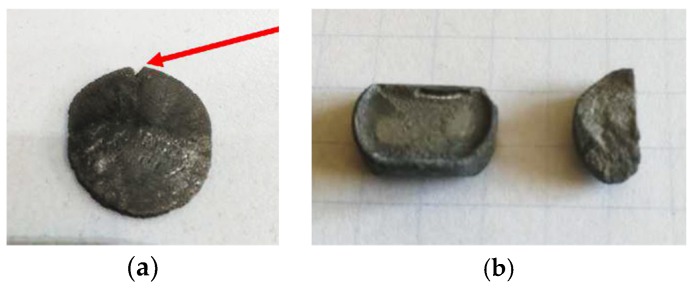
Titanium samples sintered by AC and tested by (**a**) compression with the red arrow identifying a fracture and (**b**) indirect tensile testing. The sample in (**a**) has a relative density of 71% and the samples in (**b**) have relative densities >80% (left) and <80% (right) with corresponding ductile and brittle behaviour, respectively.

Compressive and indirect tensile tests were done on the titanium samples sintered by AC. For the compressive tests, the samples exhibited a double brittle/ductile behaviour, corresponding to low and high densities. A high degree of plasticity was noticed with high densities, above 80%. Low densities showed evident cracks at the end of the compression, as shown in Figure 18a. The indirect tensile tests showed the same double nature, being both brittle and ductile. In this case, low density samples broke by brittle fracture. The IDT was estimated as 30 ± 10 MPa. High density samples fractured by shearing, and the IDT principle was therefore not applied due to the deviation from linear elastic deformation until fracture, which is a requirement for the test [30]. Titanium discs sintered by MFDC show similar mechanical trends as a function of the obtained relative density (ongoing work by the present authors).

## 5. Conclusions

An analysis of the main parameters led to the definition of the process and product fingerprints for ESF being identified, which corresponds to the most influencing process parameters and final sample properties. The process fingerprints identified were the current and compaction pressure profiles. In terms of current input, both an alternating current (AC) and middle-frequency direct current (MFDC) were analysed. ESF was possible with both machines. However, the AC machine had the disadvantage of overheating the sample, with high current peaks required to deliver a given *RMS* current. 

The product fingerprints identified were the density, microstructure, and mechanical properties in terms of the hardness and strength. Density is a main parameter and increases with increasing process energy, but melting should be avoided to minimize undesired effects, such as closed porosities, oxidation, and possible grain growth. The increase in density was greatly influenced by higher values of currents. The electrical current was therefore considered as the most valuable process fingerprint for ESF. Microstructure analysis revealed whether samples were involved in particle bonding or melting.

By testing a different geometry, namely a ring, the process was affected more by the compaction itself, generating different grades of density in symmetric regions of the rings. This was provoked by the different temperature achieved because of the non-uniform porosity distribution, involving different values of electrical resistances and therefore not the same Joule heat value. Future work should focus on force sensors as a process fingerprint to monitor the uniformity of the compaction pressure while the green body is formed. 

Additional product fingerprints can be defined for specific purposes, e.g., eccentricity would be a key product fingerprint for applications in rotors or other equipment involving high-speed rotation. General dimensional and geometrical features can also be selected. Future work should focus on a statistical analysis to establish robust correlations between process fingerprints and specific product fingerprints for given applications.
